# Increase Dietary Fiber Intake Ameliorates Cecal Morphology and Drives Cecal Species-Specific of Short-Chain Fatty Acids in White Pekin Ducks

**DOI:** 10.3389/fmicb.2022.853797

**Published:** 2022-04-07

**Authors:** Yongsheng Hao, Zhanqing Ji, Zhongjian Shen, Youjia Xue, Bo Zhang, Daxin Yu, Tong Liu, Dawei Luo, Guangnan Xing, Jing Tang, Shuisheng Hou, Ming Xie

**Affiliations:** State Key Laboratory of Animal Nutrition, Institute of Animal Sciences, Chinese Academy of Agricultural Sciences, Beijing, China

**Keywords:** dietary fiber, barrier function, microbiota, short-chain fatty acids, dietary intervention, ducks

## Abstract

The current study was to investigate the modulatory effects of total dietary fiber (TDF) levels on cecal morphology and the response of microbiota to maintain gut health for duck growth. A total of 192 14-day-old male white Pekin ducks were randomly allocated to three dietary groups and fed diets, containing 12.4, 14.7, and 16.2% TDF, respectively, until 35 days under the quantitative feed intake. Each dietary group consisted of eight replicate cages of eight birds. The results revealed that 14.7 and 16.2% TDF groups significantly promoted growth performance and improved villus height, the ratio of villus to crypt, muscle layer thickness, and goblet cells per villus of cecum in ducks. qPCR results showed that the transcriptional expression of *Claudin-1*, *Muc2*, *IGF-1*, and *SLC16A1* was significantly upregulated in cecum in 14.7 and 16.2% TDF groups. Meanwhile, the concentration of IGF-1 in circulating was significantly increased in 14.7 and 16.2% TDF groups while that of DAO was significantly decreased in 16.2% TDF group. Furthermore, the concentrations of butyrate, isobutyrate, valerate, and isovalerate in cecum were conspicuously improved in 14.7 and 16.2% TDF groups while that of propionate was significantly decreased. In addition, the concentrations of butyrate, isobutyrate, valerate, and isovalerate in cecum presented negative correlations with the concentration of DAO in circulating. 16S rRNA gene sequencing results showed that the 14.7% TDF group importantly elevated the microbial richness. Simultaneously, butyrate-producing bacteria like the family Lachnospiraceae, Oscillospiraceae, and Erysipelatoclostridiaceae were enriched as biomarkers in the 16.2% TDF group. Correlation network analysis revealed that the associations between specific bacteria and short-chain fatty acids (SCFAs) induced by different TDF levels, and the correlations among bacteria were also witnessed. For example, the genus *Monoglobus* and *CHKCI002* showed a positive correlation with butyrate, and there was a positively coexistent association between *Monoglobus* and *CHKCI002*. In summary, these data revealed that increasing the TDF level could enhance the cecal morphology and drive cecal species-specific of SCFAs in ducks.

## Introduction

Total dietary fiber consists of both non-starch polysaccharides and non-carbohydrates, including pectins, cellulose, hemicellulose, β-glucans, fructans, oligosaccharides, and starches, which cannot be hydrolyzed by endogenous enzymes in the small intestine (Cantu-Jungles and Hamaker, 2020; Pu et al., 2020). Although not fully digested, dietary fiber can influence the physicochemical characteristics of content in the gastrointestinal tract and promote novel benefits for the digestive system with promoting proper gastrointestinal architecture, maintaining microecological niche, improving the activities of digestive enzymes, and enhancing the development and integrity of the intestinal mucosa, particularly given the reduction in antibiotics use worldwide (Brownlee, 2011; Gamage et al., 2018; Wang et al., 2021). Besides, dietary fiber as prebiotic is usually fermented by microbiota, inhabiting the hindgut to produce short-chain fatty acids (SCFAs), predominantly acetate, propionate, and butyrate (Janssen and Kersten, 2015). It is known that potential value in the use of fiber supplement diet is growing for poultry. Studies reported that supplemental dietary fiber in diet could promote growth performance, intestinal morphology, and nutrients availability for ducks (Han et al., 2016, 2017; Qin et al., 2019). Despite these pieces of research illustrating the benefits of dietary fiber for duck, little to no research has been conducted on knowledge about the response of microbiota driven by dietary fiber to maintain gut health for duck growth, given that the fermentable ability in the hindgut of duck is conspicuous.

Trillions of microbes inhabit the gastrointestinal tract of poultry, which play pivotal roles in poultry health and growth, including hydrolyzing and fermenting carbohydrates, synthesizing antimicrobial compounds, lessening pathogens colonization, maintaining intestinal morphology, and regulating energy metabolism (Kogut, 2019). The majority of bacteria associated with ducks have been found in the cecum, where a relatively lower oxygen partial pressure and decreased enzyme and bile salt concentrations create conditions suitable for increasing bacterial loads, aiding indigestion and uptake of crucial nutrients (Gabriel et al., 2006; Best et al., 2016). Most of the bacteria identified by 16S rRNA community analysis belong to the phyla Bacteroidota and Bacillota (Vasai et al., 2014), which underpin the association with gut microbiome in ducks and have been linked to health states of individuals (Angelakis and Raoult, 2010). Studies on microbiology show strains of the phyla Bacteroidota and Bacillota in the cecum of ducks are mainly obligate anaerobes that are capable of decomposing uric acid and dietary fiber (Rehman et al., 2007; Wei et al., 2013; Vasai et al., 2014). Ducks lack digestive enzymes to hydrolyze dietary fiber, and thus dietary fiber becomes the main substrate for microbiota fermentation in the distal part of the gut (Montagne et al., 2003). Of major end-products from fermenting dietary fiber, SCFAs could provide energy to the epithelial cells that line the intestine (Schroeder and Backhed, 2016). Recently, Qin et al. (2020) have reported that supplemental dietary fiber in diet could promote growth performance for ducks *via* changing microbial composition, enhancing the concentrations of SCFAs subsequently and improving the barrier function. However, very little research has been conducted on the correlation between microbes and metabolites induced by dietary fiber for intestinal health in ducks when ducks are fed at different levels of dietary fiber, especially the bacterial composition being species-specific, depending on the levels of dietary fiber that is the main bacterial fuel.

Chinese annual production of meat duck is a major contributor to duck production in the world. In duck production, environmental stress and pathogens impress huge pressure on intestinal homeostasis in ducks, resulting in intestinal morphology atrophy and inflammatory response (Bai et al., 2019; Kogut, 2019). Optimal gut health is of vital importance to the performance of production animals and is synonymous in animal production industries with animal health (Kogut, 2019). Previously, through feeding white Pekin ducks with diets containing 12.4, 14.7, and 16.2% total dietary fiber (TDF), respectively, we found that increasing TDF levels significantly promoted growth performance in ducks with the bloom of SCFAs-producing bacteria and enhanced cecal morphology (Hao et al., 2021). With the enriched relative abundance of SCFAs-producing bacteria, SCFAs as metabolites of the bacteria can be passively absorbed by epithelial cells through the Na^+^-coupled monocarboxylate transporter SLC5A8 and the H^+^-coupled low-affinity monocarboxylate transporter SLC16A1 to supply energy for intestinal epithelial proliferation (den Besten et al., 2013; Koh et al., 2016). In addition, the co-occurrence of bacteria in the hindgut is important for metabolic functions, including energy metabolism associated with SCFAs (Huang et al., 2018). Moreover, Wang et al. (2020) reported that supplementation of dietary fiber in diet could enhance plasma IGF-1 concentration that was associated with the integrity of intestine in piglets. Collectively, based on our previous observations, the current research was performed to decipher the modulatory effects of TDF levels on cecal morphology and the response of microbiota to maintain gut health for duck growth under the quantitative feed intake.

## Materials and Methods

### Experimental Design and Bird Management

The dose-response experiment with 3 TDF levels (12.4, 14.7, and 16.2%) was conducted with 14-day-old male white Pekin ducks. A total of 210 1-day-old male white Pekin ducklings were fed with a commercial starter diet containing 12.12 MJ metabolizable energy/kg and 200-g crude protein/kg of diet until 14 days of age. At 14 days of age, all birds were weighed individually, and the birds with the lowest or highest body weight were removed, and 192 birds were selected finally from the remaining birds. Afterward, these ducks were allotted to 24 cages of eight birds according to similar cage weight. Each dietary treatment consisted of 8 cages with one cage as a replicate. The experimental diets were fed from 14 to 35 days of age. All ducks had free access to water, and lighting was continuous. The feed intake of ducks was quantitative, and average daily feed intake per duck is shown in Supplementary Figure 1. The temperature was kept at 33°C from 1 to 3 days of age, and then it was reduced gradually to approximately 25°C until 14 days of age and was kept at approximately 16–22°C during the growing period from 14–35 days of age.

### Experimental Diets

Experimental diets with low, medium, and high TDF levels were formulated, and the feed composition of all these diets was provided in Table 1. All experimental diets were cold-pelleted at room temperature, and the TDF levels of these diets also were analyzed according to the method of AOAC International (2007). The analyzed TDF levels of these three experimental diets were 12.4, 14.7, and 16.2%, respectively.

**TABLE 1:** Ingredients and composition of the experiment diets (%, on a dry matter basis).

Item	Content (%)		
	Low TDF diet	Medium TDF diet	High TDF diet
Corn	66.36	66.49	66.62
Soybean meal	28.10	14.05	0.00
Isolated soybean protein	0.00	6.00	12.00
Soybean dietary fiber	0.00	6.35	12.70
Soy oil	1.50	2.75	4.00
*DL*-Methionine	0.14	0.18	0.21
*L*-Lysine-HCl	0.00	0.04	0.07
*L*-Threonine	0.00	0.07	0.13
*L*-Tryptophan	0.00	0.04	0.07
Dicalcium phosphate	1.50	1.55	1.60
Limestone	1.10	1.20	1.30
Salt	0.30	0.30	0.30
Vitamin-mineral premix^a^	1.00	1.00	1.00
**Calculated nutrient levels**			
ME (Mcal/kg)	2.92	2.91	2.90
Crude protein,%	17.67	17.69	17.70
Lysine,%	0.90	0.89	0.87
Methionine,%	0.41	0.41	0.41
Methionine + cystine,%	0.70	0.63	0.56
Tryptophan	0.22	0.22	0.21
Threonine	0.73	0.73	0.72
Calcium,%	0.80	0.80	0.80
Total phosphorus	0.61	0.58	0.55
Non-ohytate phosphorus	0.39	0.39	0.39
**Analyzed value**			
TDF,%	12.4	14.7	16.2

*^a^Supplied per kilogram of total diet: Cu, 8 mg; Fe, 60 mg; Zn, 60 mg; Mn, 100 mg; Se, 0.3 mg; I, 0.4 mg; choline chloride, 1,000 mg; vitamin A, 4,000 IU; vitamin D3, 2,000 IU; vitamin E, 20 IU; vitamin K3, 2 mg; thiamin, 2 mg; riboflavin, 10 mg; pyridoxine hydrochloride, 4 mg; cobalamin, 0.02 mg; calcium-d-pantothenate, 20 mg; nicotinic acid, 50 mg; folic acid, 1 mg; and biotin, 0.15 mg. TDF, total dietary fiber.*

### Sample Collection

At 35 days of age, the body weight (BW) and average daily gain (ADG) of ducks from each cage were measured. Afterward, two ducks were randomly selected from each cage to be bled by wing vein after 11 h of fasting and then euthanized by CO_2_ inhalation. The blood samples were centrifuged at 3,500 × *g* for 15 min at 4°C. After centrifugation, the serum samples were collected and stored at –20°C. The two ceca from each selected duck were isolated. Cecal content was collected from one cecum for microbiota and SCFAs analysis, and mucosa was scraped by sterile blade after ice-cold saline flush for gene expression analysis, and all of these samples were frozen at –80°C until analyzed. And the middle section of two centimeters in length of the other cecum was collected and fixed in 4% paraformaldehyde solution for intestinal histology analysis.

### Intestinal Histology Analysis

The samples of cecum fixed in 4% paraformaldehyde solution were embedded in paraffin to generate 5-μm transverse sections and stained with Alcian blue (Servicebio Technology Co., Ltd., Wuhan, China) for microscopic examination. The sections were pictured by a Sony Alpha6000 APS camera. Then, villus height (VH), crypt depth (CD), muscle layer thickness (MLT), and the number of goblet cells per villus of cecum were determined by Image Pro-Plus 6.0 software (Media Cybernetics, Bethesda, MD, United States).

### Short-Chain Fatty Acids Analysis

The concentrations of SCFAs in cecal content were measured by using a gas chromatography system (VARIAN CP-3800, Varian, Palo Alto, CA, United States; Capillary Column 30-m- ×−250-μm- × −0.25-μm film thickness) following the previous method (Hao et al., 2021). For samples, 0.5-g cecal content (stored at –80°C) was weighted approximately. Then, 2-ml ultrapure water was added. After the vortex, each sample was centrifuged (10,000 × g) at 4°C for 15 min. The supernatant (900 μl) was then mixed with prepared 100-μl ice-cold 25% (w/v) metaphosphoric acid solution (Sinopharm Group Chemical Reagent Co., Ltd., Beijing, China) at 4°C for 4 h incubation in a shaded environment. Then, the mixture was centrifuged (10,000 × g) at 4°C for 15 min, and the solution was filtered with 45-μm nylon microporous membrane by a syringe. Finally, 1 μl of the filtrate was analyzed by GC system (N_2_ as carrier gas at 2.5 Mpa, 0.8 ml/min). The temperature of an FID detector was 280°C and that of a column heated from 60 to 220°C at a rate of 20°C min^–1^.

### Plasma Biochemical Analysis

The concentrations of triglyceride (TG), high-density lipoprotein cholesterol (HDLC), low-density lipoprotein cholesterol (LDLC), and glucose (GLU) were measured by the spectrophotometric method, using an automatic analyzer (Hitachi 7080, Tokyo, Japan) with available commercial kits (Maccura, Chengdu, China), following the standard procedure. The levels of insulin-like growth factor-1 (IGF-1), diamine oxidase (DAO), endotoxins, tumor necrosis factor-α (TNF-α), interleukin-1β (IL-1β), interleukin-10 (IL-10) were measured using ELISA commercial kits (Mosak Biotechnology Co., Ltd., Wuhan, China), and the specific operations were conducted as per the instructions of kits, and all the assays were conducted in duplicate.

### 16S rRNA Gene Amplicon Sequencing and Bioinformatic Analysis

For cecal content samples, microbial genomic DNA was extracted using QIAamp DNA Stool Mini Kit (Qiagen, Hilden, Germany), following the instructions of kits. Then, NanoDrop Spectrophotometer (Thermo Scientific, Wilmington, NC, United States) was used to detect DNA concentration, and agarose gel electrophoresis was performed to detect DNA quality. DNA concentration of each sample was diluted to 10 ng/ul using ddH2O. The specific primers (338F: 5′-ACTCCTACGGGAGGC AGCAG-3′; 806R: 5′-GGACTACHVGGGTWTCTAAT-3′) were performed to amplify the V3-V4 region of 16S rDNA. Purified amplicons were pooled in equal amounts and paired-end sequenced (2 × 250 bp) on an Illumina MiSeq platform at Majorbio Bio-Pharm Technology Co., Ltd. (Shanghai, China).

Demultiplexed raw sequencing reads were filtered with average Phred scores lower than 20 using a QIIME2 pipeline (version 1.9.1) (Caporaso et al., 2010). The potential chimeric sequences were discarded using Uchime algorithm (Edgar et al., 2011). The available sequences were clustered into operational taxonomic units (OTUs) according to 97% similarity against the SILVA database. α- and β-diversity analysis was performed using R package “Vegan.” Principal coordinate analysis (PCoA) was performed using unweighted Unifrac distance. ANOSIM based on unweighted Unifrac distance was employed to estimate distinction of microbial communities using R package “Vegan.” The relative abundance of bacteria at phyla, family, and genus levels was analyzed by one-way ANOVA to identify significantly different bacteria among groups using R package “Stats.” The heatmap of the relative abundance of bacteria at phyla, family, and genus was performed using R package “Vegan.” The linear discriminant analysis (LDA) effect size (LEfSe) algorithm was performed using the non-parametric factorial Kruskal–Wallis sum-rank test with *p* < 0.05. LDA score (>2.5) was to examine difference in abundance.

### Correlation Network Analysis

Pairwise correlation analysis was conducted between the top 100 relative abundance genus (| ρ| > 0.5, *p* < 0.05). The other pairwise correlation analysis was conducted between the top 100 relative abundance genus and SCFAs (| ρ| > 0.3, *p* < 0.05). Spearman correlation coefficients were determined using “Hmisc” R package. Correlation networks were constructed using Cytoscape software (version 3.6.1).

### RNA Isolation and Gene Expression Analysis

Total RNA was isolated from the frozen cecal mucous membrane using TRIzol reagent (Takara Biotechnology Co., Ltd., Dalian, China). The integrity of RNA was detected by electrophoresis on a 1.5% agarose gel, and the concentration and quality were measured by ultraviolet spectrophotometry using a NanoDrop 2000 (Thermo Scientific, Wilmington, NC, United States). After RNA extraction, 1,000 ng of total RNA was reverse-transcribed into cDNA using the PrimeScript RT Reagent Kit (Takara Biotechnology Co., Ltd., Dalian, China). The option Monitor 3 real-time PCR detection system (Bio-Rad) using the SYBR Green Supermix (TaKaRa) implemented real-time quantitative PCR. All primers were commercially synthesized and purified by Tsingke Biotech Co., Ltd., and are shown in Supplementary Table 1. The PCR reaction system performed in a volume of 10 μl, comprising of 1 μl of 5-time diluted cDNA, 0.4 μl each of 10-μM forward and reverse primers, 5-μl TB Green Premix Ex Taq II (Takara), 0.2-μl ROX Reference Dye II (Takara), and 3-μl DNase Free dH_2_O. Cycling conditions were as follows: 95°C for 30 s, followed by 40 cycles at 95°C for 5 s, 60°C for 34 s, under melt curve conditions at 95°C for 15 s, 60°C for 1 min, and then 95°C for 15 s (temperature change velocity, 0.5°C/s). The gene β*-actin* was selected as a reference gene, which was used to normalize the relative expression of genes of interest by the 2^–ΔΔ*CT*^ method.

### Correlogram Analysis

The correlogram assay of the environmental factors, including the expression of *Muc2*, *ZO-1*, and *IGF-1* in cecal mucosa, SCFAs in cecum, and IGF-1 and DAO in plasma, was conducted with Pearson’s correlation coefficient using “Corrgram” R package.

### Statistical Analysis

The results were analyzed using the one-way ANOVA procedure of SAS 9.4 software (SAS Institute, Inc.) followed by Duncan’s test for multi-group comparisons, with a cage as the experimental unit for analyzing growth performance and each selected bird as the experimental unit for other parameters. All results were presented as means and pooled SEM.

## Results

### Growth Performance

The effects of total dietary fiber (TDF) on body weight (BW) and average daily gain (ADG) of white Pekin ducks are shown in Table 2. The BW of birds at 35 days significantly increased (*p* < *0.05*) with increasing TDF levels. Compared with the 12.4% TDF group, the 14.7 and 16.2% TDF group had significantly ameliorated (*p* < 0.05) the ADG of birds at 35 days.

**TABLE 2:** Effects of total dietary fiber on growth performance of white Peking ducks on Day 35.

Item	12.4% TDF	14.7% TDF	16.2% TDF	SEM	*P-*value
BW (g)	2318^c^	2356^b^	2400^a^	9.34	<0.01
ADG, g	81.6^c^	83.4^b^	85.4^a^	0.45	<0.01

*^a–c^Means with different superscripts in the same row differ significantly (n = 8; p < 0.05). 12.4% TDF, 12.4% total dietary fiber; 14.7% TDF, 14.7% total dietary fiber; 16.2% TDF, 16.2% total dietary fiber; BW, body weight; ADG, average daily gain.*

### Intestinal Histology

**FIGURE 1:**
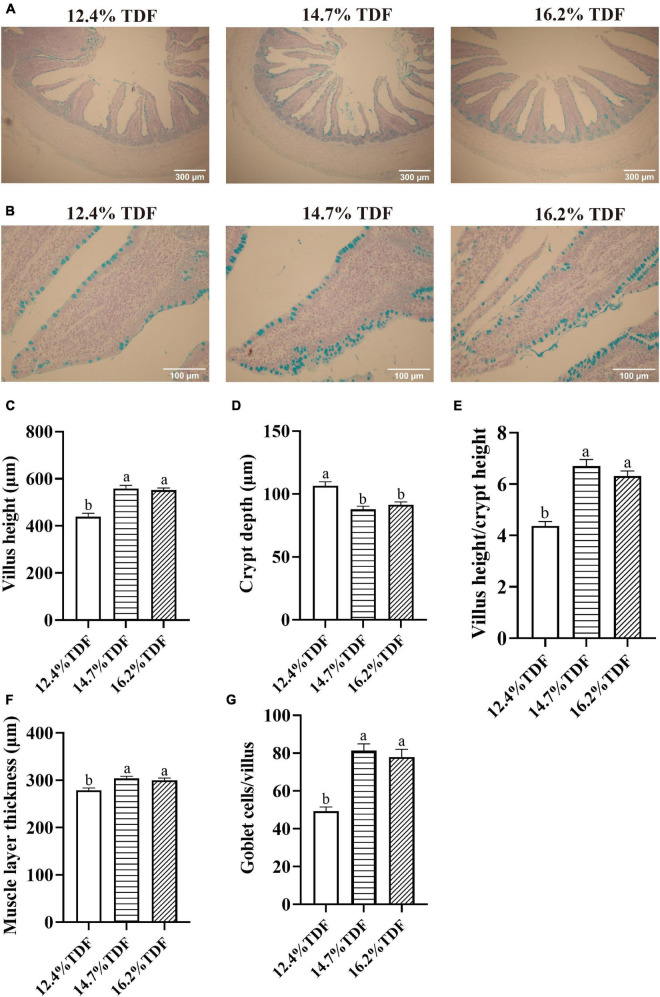
Effects of total dietary fiber on cecal morphology of white Pekin ducks on Day 35. Panel **(A)** was images of alcian blue staining in the cecum at 100 × magnification (scale: 300 μm); Panel **(B)** was images of alcian blue staining about goblet cells per villus in the cecum at 400 × magnification (scale: 100 μm); Panels **(C–G)** were villus height, crypt depth, villus height/crypt depth, muscle layer thickness, goblet cells per villus in the cecum, respectively. *^a,b^*Means with different superscripts in the same index differ significantly (*n* = 8, *p* < 0.05). 12.4% TDF, 12.4% total dietary fiber; 14.7% TDF, 14.7% total dietary fiber; 16.2% TDF, 16.2% total dietary fiber.

The effects of TDF on cecal morphology of white Pekin ducks are shown in Figure 1. The 14.7 and 16.2% TDF diets significantly elevated (*p* < 0.05) villus height (VH), the ratio of villus height to crypt depth (V/C), and muscle layer thickness (MLT) in the cecum of birds. The 12.4% TDF diet significantly increased (*p* < 0.05) the crypt depth (CD) in the cecum of birds compared with 14.7 and 16.2% TDF. The birds fed 14.7 and 16.2% TDF diets had much more (*p* < 0.05) goblet cells per villus than the birds fed 12.4% TDF diets.

### Short-Chain Fatty Acids Profiling

**FIGURE 2:**
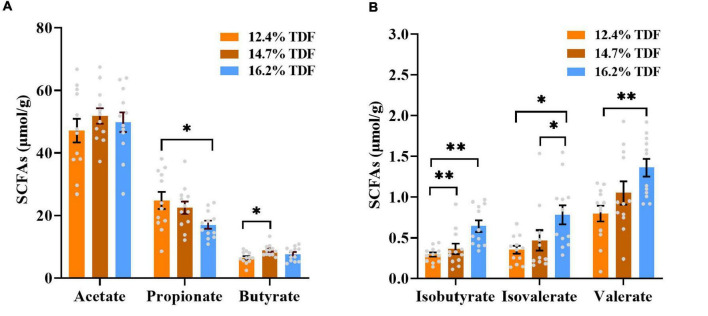
Effects of total dietary fiber on cecal short-chain fatty acids concentrations of white Pekin ducks on Day 35. Panels **(A,B)** were acetate, propionate, butyrate, isobutyrate, isovalerate, and valerate, respectively. Significant difference was recorded by 0.01 < *p* ≤ 0.05*, 0.001 < *p* ≤ 0.01^**^, *p* ≤ 0.001^***^ (*n* = 12). 12.4% TDF, 12.4% total dietary fiber; 14.7% TDF, 14.7% total dietary fiber; 16.2% TDF, 16.2% total dietary fiber; SCFAs, short-chain fatty acids.

The effects of TDF on the concentrations of cecal short-chain fatty acids of white Pekin ducks are shown in Figure 2. The diets containing 16.2% TDF significantly lowered (*p* < 0.05) propionate concentration compared with the experimental diets containing 12.4% TDF. The concentration of butyrate was conspicuously higher (*p* < 0.05) in the 14.7% TDF diet than 12.4% TDF diet. As compared with 12.4% TDF diet, the 14.7% TDF diet and 16.2% TDF diet dramatically elevated (*p* < 0.05) the concentration of isobutyrate in the cecal digesta. The diets containing 16.2% TDF also significantly increased (*p* < 0.05) the concentrations of isovalerate and valerate.

### Plasma Parameters

**FIGURE 3:**
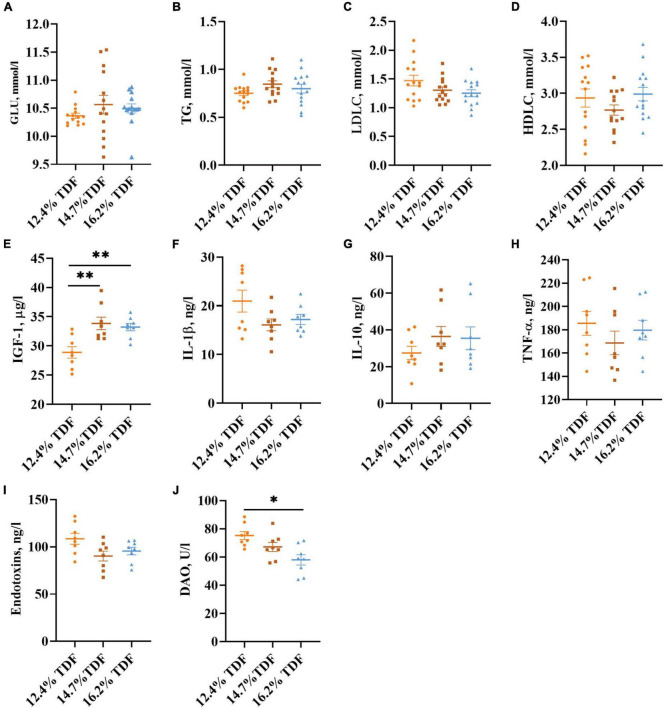
Effects of total dietary fiber on plasma parameters of white Pekin ducks on Day 35. Panels **(A–D)** were GLU, TG, LDLC, and HDLC, respectively (*n* = 14); Panels **(E–J)** were IGF-1, IL-1β, IL-10, TNF-α, endotoxins, and DAO, respectively (*n* = 8). Significant difference was recorded by 0.01 < *p* ≤ 0.05*, 0.001 < *p* ≤ 0.01^**^, *p* ≤ 0.001^***^. 12.4% TDF, 12.4% total dietary fiber; 14.7% TDF, 14.7% total dietary fiber; 16.2% TDF, 16.2% total dietary fiber; GLU, glucose; TG, triglyceride; LDLC, low-density lipoprotein cholesterol; HDLC, high-density lipoprotein cholesterol; IGF-1, insulin-like growth factor-1; IL-1β, interleukin-1β; IL-10, interleukin-10; TNF-α, tumor necrosis factor-α; DAO, diamine oxidase.

The effects of TDF on the plasma parameters of white Pekin ducks are shown in Figure 3. The diets containing 14.7% TDF and 16.2% TDF elevated (*p* < 0.05) the plasma insulin-like growth factor-1 (IGF-1) concentration of birds. Moreover, the plasma diamine oxidase (DAO) concentration of birds significantly decreased (*p* < 0.05) in the 16.2% TDF diet compared with 12.4% TDF diet.

### Gene Expression Analysis

**FIGURE 4:**
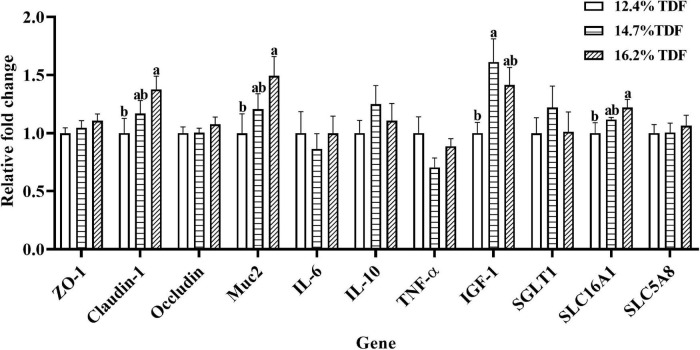
Effects of total dietary fiber on expression of mRNA levels involved in cecal barrier function and inflammatory cytokines of white Pekin ducks on Day 35. *^a,b^*Means with different superscripts in the same index differ significantly (*n* = 8, *p* < 0.05). 12.4% TDF, 12.4% total dietary fiber; 14.7% TDF, 14.7% total dietary fiber; 16.2% TDF, 16.2% total dietary fiber; ZO-1, zonula occludens-1; Muc2, mucin-2; IL-6, interleukin-6; IL-10, interleukin-10; TNF-α, tumor necrosis factor-α, IGF-1, insulin-like growth factor-1; SGLT1, Na^+^–glucose co-transporter 1; SLC5A8, Na^+^-coupled monocarboxylate transporter; SLC16A1, H^+^-coupled low affinity monocarboxylate transporter.

The effects of TDF on the expression of mRNA levels involved in cecal barrier function and inflammatory cytokines of white Pekin ducks are shown in Figure 4. As compared with 12.4% TDF diet, 14.7% TDF, and 16.2% TDF diet significantly increased (*p* < 0.05) the expression level of *Claudin-1* and *Mucin-2* (*Muc2*) in the cecal mucosa. The expression level of *IGF-1* was distinctly raised (*p* < 0.05) in the cecum of birds supplemented with 14.7% TDF diet. Additionally, the expression level of *H^+^-coupled low-affinity monocarboxylate transporter* (*SLC16A1*) significantly increased (*p* < 0.05) in the 16.2% TDF diet.

### Cecal Microbiota Analysis

**FIGURE 5:**
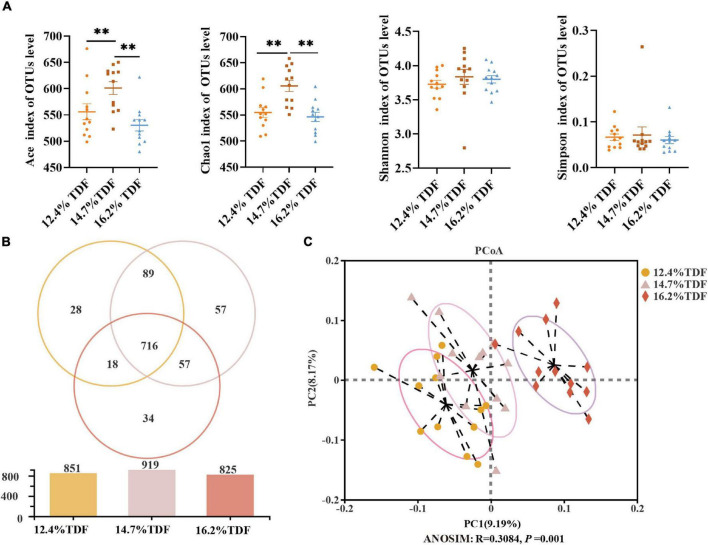
Effects of total dietary fiber on cecal microbial diversity of white Pekin ducks on Day 35. Panel **(A)** was α-diversity index; Panel **(B)** was a Venn diagram; Panel **(C)** was β-diversity. Significant difference was recorded by 0.01 < *p* ≤ 0.05*, 0.001 < *p* ≤ 0.01^**^, *p* ≤ 0.001^***^. 12.4% TDF, 12.4% total dietary fiber; 14.7% TDF, 14.7% total dietary fiber; 16.2% TDF, 16.2% total dietary fiber.

After merging the raw data and filtering the low-quality sequences, a total of 2,105,396 clean reads were obtained from 36 samples and were clustered into 999 operational taxonomic units (OTUs) based on the criterion of 97% sequence similarity. Rare faction curves and Shannon cures were performed to examine whether the sequencing depth and data volume were sufficient (Supplementary Figure 2). To assay the α-diversity of cecal microbiota, ACE and Chao 1 were employed for the richness of microbial community, and Shannon and Simpson were examined for diversity of the microbial community. As compared with 12.4 and 16.2% TDF diets, 14.7% TDF diet conspicuously elevated (*p* < 0.05) ACE and Chao 1 estimators, while there were no significant differences in Shannon and Simpson indices (Figure 5A). As shown in Figure 5B, there were 716 common OTUs in three different levels of TDF treatment, and 12.4, 14.7, and 16.2% TDF treatment covered individual 28, 57, and 34 OTUs, respectively. To further evaluate the microbial profile, PCoA based on unweighted Unifrac distance was performed to illustrate that birds-fed three levels of dietary fiber diet were distributed to three unique clusters in Figure 5C (ANOSIM: R = 0.308, *p* < 0.05).

**FIGURE 6:**
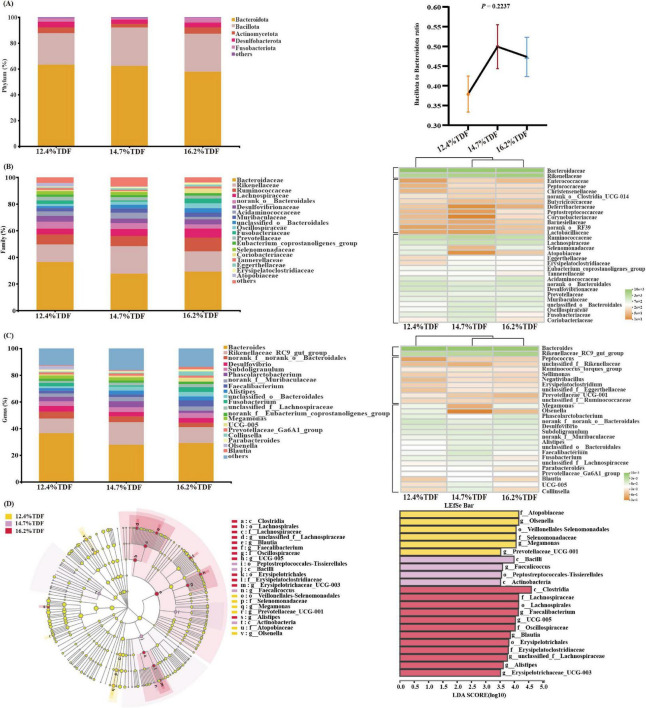
Effects of total dietary fiber on cecal microbial composition of white Pekin ducks on Day 35. Panels **(A–C)** were microbiota composition at phylum, family, and genus levels, respectively; Panel **(D)** was linear discriminant analysis effect size (LEfSe) with LDA > 2.5, *p* < 0.05.

The relative taxa abundance of microbiota was analyzed at phylum, family, and genus taxonomic levels. At the phylum level, Bacteroidota, Bacillota, Desulfobacterota, Actinomycetota, and Fusobacteriota were the main phyla (>1%) in the cecal community of ducks, and the ratio of Bacillota to Bacteroidota was increased when the birds were fed a higher TDF diet (Figure 6A). At the family level, the sequences representative of Bacteroidaceae, Rikenellaceae, Ruminococcaceae, and Lachnospiraceae dominated the cecal microbial community (Figure 6B). The relative abundance of Erysipelatoclostridiaceae and Christensenellaceae in the 14.7 and 16.2% TDF diet was higher (*p* < 0.05) than that in the 12.4% TDF diet, whereas the relative abundance of Selenomonadaceae, Deferribacteraceae, and Bifidobacteriaceae in 16.2% TDF diet was lower (*p* < 0.05) than that in the 12.4 and 14.7% TDF diet (Supplementary Figure 3A). At the genus level, the bird’s cecal microbiota of three groups was predominated by *Bacteroides* and *Rikenellaceae_RC9_gut_group* (Figure 6C). Compared with the 12.4% TDF group, the sequences of *Alistipes*, *UCG-005*, *Blautia*, *Negativibacillus*, *Christensenellaceae_R7_group*, and *Erysipelotrichaceae_UCG_003* were significantly ameliorated (*p* < 0.05) when the birds were fed 16.2% TDF diet, while the sequences of *Megamonas* and *Mucispirillum* were dramatically lower (*p* < 0.05) in the 16.2% TDF group (Supplementary Figure 3B).

In addition, the LEfSe method was performed to identify the significant phylotypes relating to differentiation among groups in Figure 6D. The sequences of class Bacilli and Actinobacteria were enriched in the 14.7% TDF group, whereas that of class Clostridia were characterized in the 16.2% TDF group. At a family level, Lachnospiraceae (*unclassified_f_ Lachnospiraceae* and *Blautia*), Oscillospiraceae (*UCG-005*), and Erysipelatoclostridiaceae (*Erysipelatoclostridiaceae_UCG-005*) were found to be characterized in the 16.2% TDF group. Besides, Selenomonadaceae (*Megamonas*) and Atopobiaceae (*Olsenella*) were observed to be enriched as biomarkers in the 12.4% TDF group.

### Correlation Network Analysis Between Microbiota and Short-Chain Fatty Acids

**FIGURE 7:**
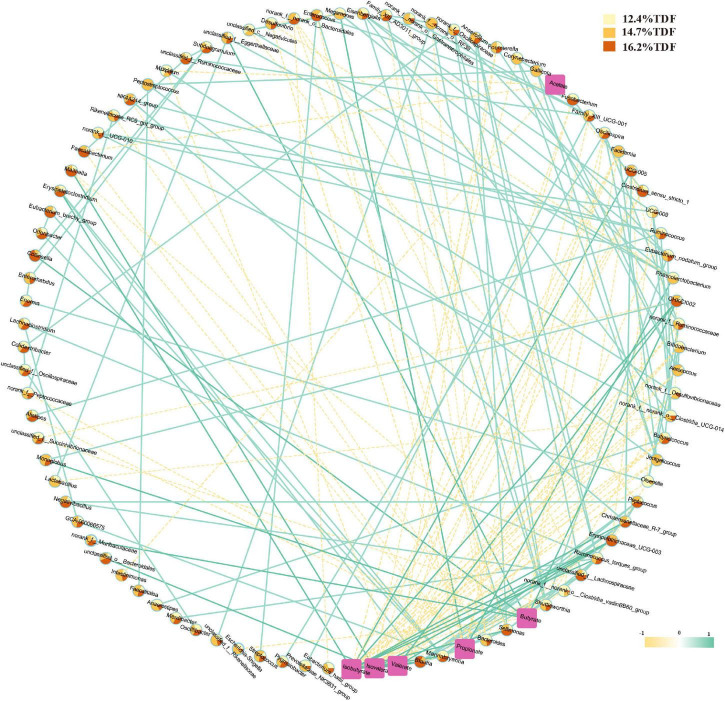
Correlation network between cecal microbiota and SCFAs. A pairwise Spearman correlation analysis was conducted between the top 100 relative abundance genus (| ρ| > 0.5, *p* < 0.05). The other pairwise Spearman correlation analysis was conducted between the top 100 relative abundance genus and SCFAs (| ρ| > 0.3, *p* < 0.05). The relative abundance genus among groups was shown in color-coded dots. A positive and negative correlation is presented by a solid light blue line and a dash light orange line, respectively. The intensity of the color denotes the strength of the correlation. About 12.4% TDF, 12.4% total dietary fiber; 14.7% TDF, 14.7% total dietary fiber; 16.2% TDF, 16.2% total dietary fiber.

Pairwise correlation assay identifies specific associations between microbiota at the genus level and SCFAs with 94 nodes and 200 edges in Figure 7. According to the network, *Clostridium_sensu_stricto_1*, *Faecalibacterium*, *Oscillospira*, and *Intestinimonas* negatively correlated with acetate. *Megamonas* and *Phascolarctobacterium* showed a positive correlation with propionate, and there was a positive correlation between *Megamonas* and *Phascolarctobacterium*, and *Faecalibacterium* was negatively associated with propionate. *Monoglobus*, *unclassified_f_Eggerthellaceae*, *Collinsella*, *norank_f_Muribaculaceae*, and *CHKCI002* were positively associated with butyrate, and *Monoglobus* and *CHKCI002* were observed as a positive association between each other, and *Facklamia*, *Oscillospira*, and *Escherichia-Shigella* showed a negative association with butyrate. Besides, *Blautia*, *CHKCI002*, *Erysipelotrichaceae_UCG-003*, *Marvinbryantia*, and *UCG-005* were positively correlated with isobutyrate, and there was a positive association between *Blautia* and *Marvinbryantia*. *Erysipelotrichaceae_UCG-003*, *CHKCI002*, and *Blautia* presented a positive correlation with valerate, and *Erysipelotrichaceae_UCG-003* and *Blautia* were also observed as a positive association with isovalerate.

### Correlogram Analysis

**FIGURE 8:**
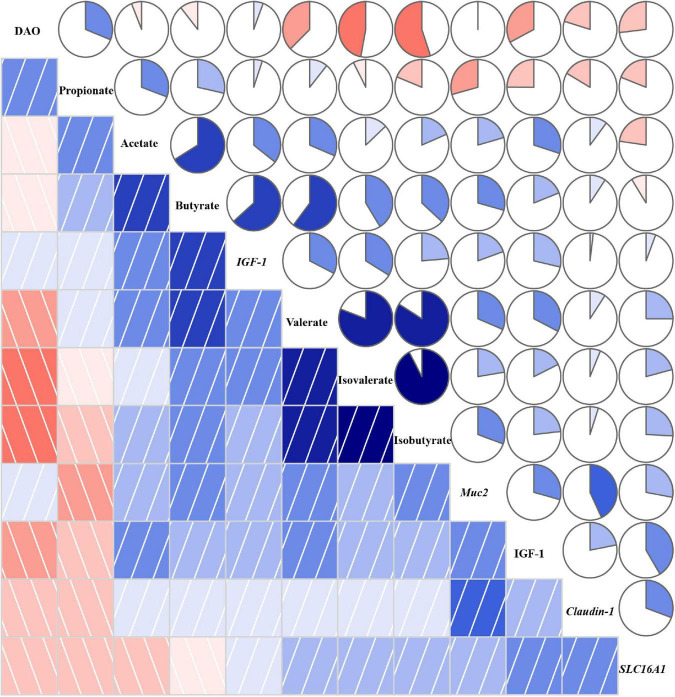
Correlation coefficient matrix between environmental factors. A pairwise Pearson correlation analysis was conducted between environmental factors to analyze the relationship. A positive and negative correlation is presented by blue and red, respectively. The intensity of the color denotes the strength of the correlation. DAO, diamine oxidase; IGF-1, insulin-like growth factor-1; Muc2, mucin-2; SLC16A1, H^+^-coupled low-affinity monocarboxylate transporter. About 12.4% TDF, 12.4% total dietary fiber; 14.7% TDF, 14.7% total dietary fiber; 16.2% TDF, 16.2% total dietary fiber.

The correlation coefficient matrix assay between environmental factors is shown in Figure 8. There was a negative correlation between DAO and SCFAs except for propionate, and IGF-1 in plasma also witnessed the same correlation. The expression of *IGF-1* showed a positive association with SCFAs, especially butyrate, valerate, and isovalerate, and the expression of *Muc2 and Claudin-1* was also positively associated with SCFAs except for propionate. The expression of *SLC16A1* showed a positive correlation with isobutyrate, isovalerate, and valerate. Besides, propionate was negatively associated with isobutyrate, isovalerate, and valerate. The expression of *IGF-1* was positively correlated with *Muc2*, *Claudin-1*, *SLC16A1*, and IGF-1.

## Discussion

The gastrointestinal tract is of vital importance for nutrient digestion and absorption, mucus secretion, and prevention of the body from pathogenic microbiota, in which barrier function and fermentability are indispensable for such vital roles (Okumura and Takeda, 2018; Zhang et al., 2020). Previous studies indicated that a high dietary fiber level could not only elevate the weight of the cecum but increase the VH, V/C, and mucosal thickness in ducks (Qin et al., 2020; Hao et al., 2021). In the current research, the amelioration in VH, V/C, and MLT of the cecum was also witnessed when ducks were fed high TDF diets. Besides, the number of goblet cells per villus increased in the higher TDF treatments. In line with our study, Paturi et al. (2012) reported that a dietary fiber diet containing inulin could increase the number of goblet cells in the colon of rats. Goblet cells could do biosynthesis, assemble, and secrete mucin, including Muc1, Muc2, and Muc4, as a disulfide cross-linked network along the crypt to villus axis of the gut, which forms the intestinal mucus barrier to prevent pathogens and toxins permeating intestinal epithelial cells (McCauley and Guasch, 2015; Desai et al., 2016). Importantly, the improved cecal morphology may be induced by a higher concentration of plasma IGF-1 in the 14.7 and 16.2% TDF groups. As known, IGF-1 conspicuously regulates intestinal growth and development, stimulating proliferation of intestinal mucosa, attenuating gut atrophy, and maintaining intestinal epithelial integrity (Chen et al., 1999; Lorenzo-Zuniga et al., 2006; Xiu et al., 2021). Wang et al. (2020) also observed that dietary fiber could ameliorate the concentration of IGF-1 in circulating blood, which was beneficial for intestinal morphology in piglets. Enhanced cecal morphology is considered to be beneficial for digestion and absorption of nutrients, and contributes to the growth and development of ducks. In our study, increasing TDF levels could promote the growth performance of ducks with the conspicuous elevation in cecal morphology. This observation supports the concept that dietary fiber in the diet can ameliorate the intestinal morphological surface area to increase the ability of digestion and absorption, and promote growth performance (Liu et al., 2018; Pu et al., 2020). These findings are adequate to illustrate that the growth promotion of dietary fiber can be partially attributed to improved intestinal morphology and function.

Dietary fiber as an important substrate for the community of microbiota inhabits in the distal gut is driving gut microbiota diversity and composition of ducks to maintain gut health, and thereby promote the growth of ducks. In the present study, data from analysis of β-diversity showed that dietary fiber could alter a microbial profile effectively (ANOSIM: R = 0.308, *p* < 0.05). Increasing ACE and Chao 1 estimators value-reflecting microbiota richness were observed in the 14.7% TDF group, which draws it into a spotlight that it is useful to balance gut microbiota population by dietary fiber. The stable niche in the gut contributes to defense against pathogens, digests complex dietary macronutrients, and maintains the immune system (Sonnenburg and Sonnenburg, 2014; Koh et al., 2016). Furthermore, Bacteroidota and Bacillota were the predominant phyla in the cecum of ducks, regardless of the changed levels of TDF in the diet, consistent with published findings on the microbial composition of ducks (Vasai et al., 2014). The increased ratio of Bacillota to Bacteroidota was also observed in the higher TDF diet, which is in line with the study of Hao et al. (2021) that high TDF diets elevated the ratio of Bacillota to Bacteroidota in the cecum of ducks. The higher Bacillota to Bacteroidota ratio in the gut microbial community of animals was considered to improve nutrient digestibility and gain body weight, and the reverse was associated with weight loss (Ley et al., 2006; Mariat et al., 2009; Singh et al., 2013; Dai et al., 2020). It is the improved relative abundance of families Ruminococcaceae and Lachnospiraceae at expense of family Bacteroidaceae that contributed to the raised Bacillota to the Bacteroidota ratio in the higher TDF group. Bacteria in the phylum Bacillota are programmed to be associated with healthy intestinal barrier function (Vital et al., 2017; Truax et al., 2018; Vujicic et al., 2018). In addition, bacteria belonging to Bacteroidota, which was the largest phylum of Gram-negative, were involved in releasing lipopolysaccharide leading to inflammation activation (Ortega-Hernandez et al., 2020). The decreased relative abundance of phylum Bacteroidota in the current research may be related to the lower inflammatory factors, including the concentrations of IL-1β and TNF-α in plasma, as well as the expression of *IL-6* and *TNF-*α in cecal mucosa, although their effects were not significant. Wang et al. (2019) reported that *Bacteroides* in the phylum of Bacteroidota showed a positive correlation with the expression of inflammatory factors *IL-1*β, *IL-8*, and *TNF-*α in the ileum of laying hens. From LEfSe, the alternation in enriched biomarkers from Atopobiaceae and Selenomonadaceae in the 12.4% TDF group to Lachnospiraceae, Oscillospiraceae, and Erysipelatoclostridiaceae in the 16.2% TDF group was also witnessed, which was concomitant with previous observations that dietary fiber could bring benefits to ameliorate the abundance of butyrate-producing microbiota to promote barrier function in the gut (Louis and Flint, 2017; van der Hee and Wells, 2021). In this sense, dietary fiber could be utilized to shape cecal microbiota composition of ducks to maintain a niche in the gut, which provides the health promotion for the growth of ducks.

Admittedly, dietary fiber could regulate the cecal microbiota on the one hand, while, on the other hand, cecal microbiota is also involved in the metabolic pathway of dietary fiber. It is established that dietary fiber is fuel for microbial fermentation in the hindgut, and the major end-products from the microbial fermentative activity are SCFAs (Liu et al., 2021). In the present study, the concentrations of butyrate, isobutyrate, valerate, and isovalerate increased with TDF levels, while that of propionate witnessed the opposite result. Theoretically, increasing dietary fiber levels could elevate the concentration of propionate in the cecum, and previous pieces of research also observed the same outcome (Pu et al., 2020; Qin et al., 2020). Furthermore, the correlation network was performed to study the interesting observation in the current research. From the network, it is obvious that genera *Megamonas* and *Phascolarctobacterium* were positively correlated with propionate, and there was a positive relationship between genera *Megamonas* and *Phascolarctobacterium*, and the relative abundance of them decreased with increasing TDF levels, by which the decreased concentration of propionate in the higher TDF diet could be explained as both of them favored propionate production *via* the succinate pathway (Sergeant et al., 2014; Koh et al., 2016). Besides, propionate could form valerate in the presence of methanol as an electron when orders Coriobacteriales and Erysipelotrichales were candidates for elongation (de Smit et al., 2019). The relative abundance of genus *Erysipelotrichaceae_UCG-003* of order Erysipelotrichales and genus *CHKCI002* of order Coriobacteriales and the concentration of valerate elevated in the 14.7 and 16.2% TDF diet, and both of them showed a positive correlation with valerate, which may be proposed as an explanation for decreased concentration of propionate in the higher TDF diet. In addition, the relative abundance of families Ruminococcaceae, Lachnospiraceae, and Oscillopiraceae, known as butyrate-producing bacteria with fibrolytic specialization (Biddle et al., 2013; Walugembe et al., 2015), increased in 14.7 and 16.2% TDF groups, which contributed to elevating the concentration of butyrate in the cecum of ducks in those groups. Concomitant with these observations, Qin et al. (2020) also demonstrated that the relative abundance of family Prevotellaceae producing butyrate increased in the higher dietary fiber diets. Genus-level network analysis found genera *Monoglobus*, and *CHKCI002* showed a positive correlation with butyrate, and there was a positively coexistent relationship between genera *Monoglobus* and *CHKCI002*. It was reported that genus *Monoglobus* as the novel phylogenetic lineage of family Ruminococcaceae had the ability to ferment dietary fiber (Goris et al., 2021), and the ability of digesting complexity polysaccharides may facilitate butyrate production. The genus *CHKCI002*, affiliating to the family Eggerthellaceae, shared a symbiotic correlation with genus *Monoglobus*, which may bring benefits for duck health (Selma et al., 2017; Low et al., 2021). Knowledge of the genus *marvinbryantia* of family Lachnospiraceae, known as butyrate-producing strain, could underpin a positive association with isobutyrate in the network, which might function as core species by sharing co-occurrence relationships with *Erysipelatoclostridium*, *Blautia*, *Sellimonas*, and *Christensenellaceae_R-7_group* to produce isobutyrate. The genus *Blautia* of family Lachnospiraceae and genus *Erysipelotrichaceae*_*UCG*_*003* of family Erysipelatoclostridiaceae as core microbiota with coexistent correlation were linked with isobutyrate, isovalerate, and valerate positively in the network, all of which may contribute to forming SCFAs by fermenting dietary fiber. Within the core, each species might abstain from competing with each other and act as key ecosystem service providers by utilizing dietary fiber as a substrate to produce SCFAs, which may, in turn, induce these species to be enriched in the host simultaneously (Park et al., 2012; Zhang et al., 2015). The acetate-producing Bifidobacterium, however, presented lower prevalence in the network, indicating the probability that this genus may not serve as a fundamental acetate-producing species *via* fermenting dietary fiber in ducks, although it was considered as a major beneficial symbiont in animal gastrointestinal tracts (Fukuda et al., 2011; Zhang et al., 2015). Therefore, microbiota fermentative activity is pivotal to produce SCFAs by degrading dietary fiber, especially coexistent correlation between bacteria.

Short-chain fatty acids are major microbial metabolites that are derived from microbial fermentation of dietary fiber in the hindgut of ducks. They can be taken up efficiently by epithelial cells *via* Na^+^-coupled monocarboxylate transporter SLC5A8 and the H^+^-coupled low-affinity monocarboxylate transporter SLC16A1 to regulate host physiology for benefits, including energy salvage, gene expression regulation, and signaling molecules recognition (Koh et al., 2016; Morrison and Preston, 2016). In the present study, the expression of the *SLC16A1* gene was conspicuously increased in the 14.7 and 16.2% TDF diet with higher concentrations of butyrate, isobutyrate, valerate, and isovalerate. Furthermore, the expression of the *SLC16A1* gene was positively correlated with isobutyrate, valerate, and isovalerate, respectively, which illustrated that SLC16A1 may be mainly associated with isobutyrate, valerate, and isovalerate transportation that supplied energy for intestinal epithelial proliferation. In addition, it is established that SCFAs, butyrate, in particular, are important substrates for regulating the integrity of barrier function in the gut (Clausen and Mortensen, 1995; Cani et al., 2008). The glycoprotein-rich mucus as the first barrier of defense against pathogens and microbes is a dynamic and chemically complex barrier composed largely of Muc2 secreted by goblet cells in the gut (Desai et al., 2016). Desai et al. (2016) found that diet deficiency in dietary fiber could initiate gut microbiota to feed on a mucus barrier, leading to pathogen susceptibility and inflammation. In the current research, the expression of *Muc2* gene in the cecum conspicuously increased in the higher TDF group with elevated concentration of butyrate, and showed a positive correlation with butyrate. Besides, the number of goblet cells per villus was also conspicuously raised in the same TDF group. The results were following the study of Wellington et al. (2020), who found that a higher dietary fiber diet could bring benefits for the expression of *Muc2* gene and the concentration of butyrate in the cecum of piglets. Paturi et al. (2012) also reported that the expression of *Muc2* gene was positively correlated with butyrate and goblet cells, respectively. Furthermore, butyrate appears to be the efficient regulator for tight junction and could enhance the expression of Claudin-1, Zonula Occludens-1 (ZO-1), and Occludin that are critical components of the tight junction (Wang et al., 2012). From our observations, butyrate presented a positive correlation with the expression of *Claudin-1*, and the expression of *Claudin-1* was significantly increased in the 14.7 and 16.2% TDF group with elevated concentration of butyrate. In line with our research, Chen et al. (2020) reported that dietary fiber elevated the expression of *Claudin-1* in the ileum of piglets partly through regulating SCFAs. In addition, there was a higher expression of the *IGF-1* gene in the cecum, and the concentration of plasma IGF-1 significantly elevated from 14.7 and 16.2% TDF groups, and both of them presented a positive correlation with the expression of *Muc2* and *Claudin-1* gene. It is known that IGF-1 can ameliorate intestinal barrier function, and thus attenuate bacterial translocation (Lorenzo-Zuniga et al., 2006). Intriguingly, these results are in accordance with the biomarker of intestinal permeability measured by blood parameters. DAO, an intestinal mucosal enzyme mainly distributed in the cytoplasm, serves as the marker of the integrity of barrier function in the gastrointestinal as hypoplasia of intestinal barrier function specifically results in releasing it into circulating blood (Thompson et al., 1987). Increasing TDF levels in diet significantly decreased the concentration of plasma DAO in the present study, which indicated that the intestinal barrier function of ducks was improved in the higher dietary fiber diets. The concentration of DAO in plasma was also negatively correlated with SCFAs, especially isobutyrate, showing that SCFAs as fermentation products of dietary fiber were important for regulating intestinal barrier function of ducks. The results are consistent with previous study that dietary fiber decreased the plasma DAO concentration with increased concentration of butyrate that facilitated barrier function in ileum of piglets (Wang et al., 2020). Collectively, these results illustrated that the improvement of cecal morphology and barrier function of ducks could be facilitated by increasing dietary TDF.

## Conclusion

In a nutshell, our results illustrate that increasing the TDF level could enhance the cecal morphology and drive cecal species-specific of SCFAs in ducks. In detail, the family Lachnospiraceae, Oscillospiraceae, and Erysipelatoclostridiaceae as butyrate-producing bacteria were enriched in the 16.2% TDF group accompanied by the improved concentration of butyrate in the cecum of ducks. Network analysis revealed the correlations between core SCFAs-producing bacteria, including the genus *Blautia*, *Erysipelotrichaceae_UCG_003*, *CHKCI002*, *Monoglobus*, *Megamonas*, and *Phascolarctobacterium*, and the response of core bacteria to SCFAs induced by the TDF level. The enhanced cecal barrier function may be facilitated by an increase in butyrate concentration as the enrichment of butyrate-producing bacteria in the cecum and the concentration of IGF-1 in circulating, all of which contributed to growth performance and intestinal health in ducks.

## Data Availability Statement

The datasets presented in this study can be found in online repositories. The names of the repository/repositories and accession number(s) can be found in the article/Supplementary Material.

## Ethics Statement

The animal study was reviewed and approved by animal care and use Committee of Institute of Animal Sciences of Chinese Academy of Agricultural Sciences. Written informed consent was obtained from the owners for the participation of their animals in this study.

## Author Contributions

YH performed the animal experiment and wrote the manuscript. YH, ZJ, ZS, YX, BZ, DY, DL, GX, and JT assisted in conducting the experiment and collecting the samples. ZJ helped to perform the qPCR experiment. SH and MX designed the experiment. MX revised the manuscript. All authors contributed to the study and supported the submitted version.

## Conflict of Interest

The authors declare that the research was conducted in the absence of any commercial or financial relationships that could be construed as a potential conflict of interest.

## Publisher’s Note

All claims expressed in this article are solely those of the authors and do not necessarily represent those of their affiliated organizations, or those of the publisher, the editors and the reviewers. Any product that may be evaluated in this article, or claim that may be made by its manufacturer, is not guaranteed or endorsed by the publisher.
