# Supplementary material for: Increase Dietary Fiber Intake Ameliorates Cecal Morphology and Drives Cecal Species-Specific of Short-Chain Fatty Acids in White Pekin Ducks
Source: Front Microbiol. 2022 Apr 7;13:853797. doi: 10.3389/fmicb.2022.853797 (PMC9021919; doi:10.3389/fmicb.2022.853797)
Supplement: Supplementary file 1 [file Data_Sheet_1.ZIP › Supplementary_Material/Supplementary_Material.docx]

Supplementary Material

# Supplementary Tables and Figures

## Supplementary Table

**Supplementary Table S1.** The primers for quantitative real-time PCR

| Gene | Gene ID | Primer | Sequence (5′-3′) | Size (bp) |
| --- | --- | --- | --- | --- |
| *ZO-1* | XM_038184905.1 | Forward | gagccttcagaccattccagaca | 155 |
|  |  | Reverse | tcgcctgccacctcttccata |  |
| *MUC2* | XM_038180256.1 | Forward | ccataagccagaccacgccatc | 143 |
|  |  | Reverse | caaggtgctcaaggtgctccag |  |
| *Occludin* | XM_013109403.1 | Forward | caggatgtggcagaggaatacaa | 160 |
|  |  | Reverse | ccttgtcgtagtcgctcaccat |  |
| *Claudin-1* | XM_013108556.4 | Forward | gaccaggtgaagaagatgcggatg | 107 |
|  |  | Reverse | cgagccactctgttgccatacc |  |
| *IGF-1* | EU031044.1 | Forward | gcagtagacgcttacaccacaa | 84 |
|  |  | Reverse | cacagtacatctccagcctcct |  |
| *IL-6* | XM_027450925.2 | Forward | tggcaacgacgataaggcagat | 138 |
|  |  | Reverse | ggaggatgaggtgtgtggtgat |  |
| *IL-10* | NM_001310368.1 | Forward | gcctccacttgtctgacctcct | 177 |
|  |  | Reverse | gcatcatctccagcaccgactg |  |
| *TNF-α* | XM_013105371.4 | Forward | tggctaagaccgtggtcagtt | 121 |
|  |  | Reverse | gcagttaggtgacgctgaatga |  |
| *SGLT1* | XM_005026696.5 | Forward | cggtggattcttcctggctgga | 114 |
|  |  | Reverse | gctgctgctgttcctgctatgc |  |
| *SLC5A8* | XM_021275685.3 | Forward | tacgagcagtggtggttcagga | 152 |
|  |  | Reverse | gccagtccatgtgaaggttcca |  |
| *SLC16A1* | XM_005013691.5 | Forward | gtctcctgctgaactgctgtgt | 154 |
|  |  | Reverse | ccaccgatgaggtctgtgctag |  |
| *β-actin* | NM_001310408.1 | Forward | ccagccatctttcttgggta | 105 |
|  |  | Reverse | gtgttggcgtacaggtcctt |  |

Muc2, Mucin-2; ZO-1, Zonula occludens-1; IGF-1, Insulin-like growth factor-1; IL-6, interleukin 6; IL-10, interleukin 10; TNF-α, Tumor necrosis factor-α; SGLT1, Na^+^–glucose co-transporter 1; SLC5A8, Na^+^-coupled monocarboxylate transporter; SLC16A1, H^+^-coupled low affinity monocarboxylate transporter.

## Supplementary Figures





**Supplementary Figure S1.** Average daily feed intake per duck under the quantitative feed intake from day15 to 35.


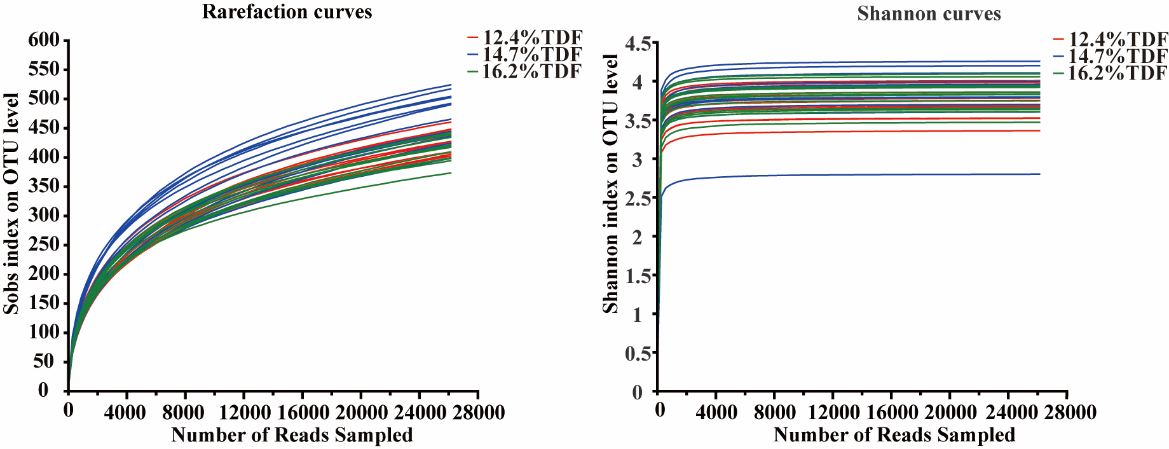


**Supplementary Figure S2.** Effects of total dietary fiber on cecal microbial rarefaction curve of White Pekin ducks on day 35. 12.4% TDF, 12.4% total dietary fiber; 14.7% TDF, 14.7% total dietary fiber; 16.2% TDF, 16.2% total dietary fiber.


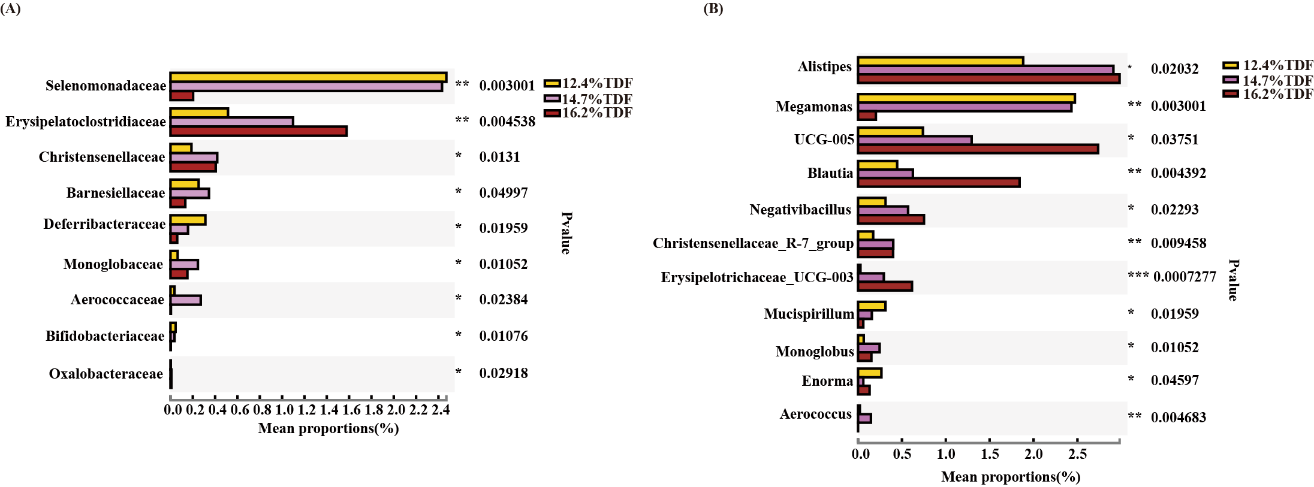


**Supplementary Figure S3.** Effects of total dietary fiber on differential species of cecal microbiota identified at family and genus levels of White Pekin ducks on day 35. (A) analysis of difference among microbiota community at family level. (B) analysis of difference among microbiota community at genus level. 12.4% TDF, 12.4% total dietary fiber; 14.7% TDF, 14.7% total dietary fiber; 16.2% TDF, 16.2% total dietary fiber.
